# Endochondral Growth Defect and Deployment of Transient Chondrocyte Behaviors Underlie Osteoarthritis Onset in a Natural Murine Model

**DOI:** 10.1002/art.39508

**Published:** 2016-03-28

**Authors:** K. A. Staines, K. Madi, S. M. Mirczuk, S. Parker, A. Burleigh, B. Poulet, M. Hopkinson, A. J. Bodey, R. C. Fowkes, C. Farquharson, P. D. Lee, A. A. Pitsillides

**Affiliations:** ^1^ Royal Veterinary College, University of London, London, UK, and Roslin Institute and Royal (Dick) School of Veterinary StudiesUniversity of Edinburgh, Easter Bush, UK; ^2^ Manchester X‐Ray Imaging Facility, University of Manchester, Manchester, UK; ^3^ Royal Veterinary College, University of London, London, UK;; ^4^ University College London Medical School, London, UK; ^5^ Diamond Light Source, Harwell Science and Innovation Campus, Didcot, UK; ^6^ Roslin Institute and Royal (Dick) School of Veterinary Studies, University of Edinburgh, Easter Bush, UK

## Abstract

**Objective:**

To explore whether aberrant transient chondrocyte behaviors occur in the joints of STR/Ort mice (which spontaneously develop osteoarthritis [OA]) and whether they are attributable to an endochondral growth defect.

**Methods:**

Knee joints from STR/Ort mice with advanced OA and age‐matched CBA (control) mice were examined by Affymetrix microarray profiling, multiplex polymerase chain reaction (PCR) analysis, and immunohistochemical labeling of endochondral markers, including sclerostin and MEPE. The endochondral phenotype of STR/Ort mice was analyzed by histologic examination, micro–computed tomography, and ex vivo organ culture. A novel protocol for quantifying bony bridges across the murine epiphysis (growth plate fusion) using synchrotron x‐ray computed microtomography was developed and applied.

**Results:**

Meta‐analysis of transcription profiles showed significant elevation in functions linked with endochondral ossification in STR/Ort mice (compared to CBA mice; *P* < 0.05). Consistent with this, immunolabeling revealed increased matrix metalloproteinase 13 (MMP‐13) and type X collagen expression in STR/Ort mouse joints, and multiplex quantitative reverse transcriptase–PCR showed differential expression of known mineralization regulators, suggesting an inherent chondrocyte defect. Support for the notion of an endochondral defect included accelerated growth, increased zone of growth plate proliferative chondrocytes (*P* < 0.05), and widespread type X collagen*/*MMP‐13 labeling beyond the expected hypertrophic zone distribution. OA development involved concomitant focal suppression of sclerostin/MEPE in STR/Ort mice. Our novel synchrotron radiation microtomography method showed increased numbers (*P* < 0.001) and mean areal growth plate bridge densities (*P* < 0.01) in young and aged STR/Ort mice compared to age‐matched CBA mice.

**Conclusion:**

Taken together, our data support the notion of an inherent endochondral defect that is linked to growth dynamics and subject to regulation by the MEPE/sclerostin axis and may represent an underlying mechanism of pathologic ossification in OA.

Osteoarthritis (OA) is a degenerative joint disease and a health care burden throughout the world. Characterized by articular cartilage loss, subchondral bone thickening, and osteophyte formation, OA causes much pain and disability. Its underlying molecular mechanisms are, nevertheless, not fully understood; indeed, even the precipitating pathology is still a matter of debate. As such, there is an ever‐growing need for an effective disease‐modifying treatment. Canine hip dysplasia is a hereditary predisposition to the development of degenerative OA and is more common in certain breeds, in particular larger breeds which tend to grow more rapidly 1. While no direct link has been made between growth dynamics and OA, recent murine and human studies have prompted speculation that articular cartilage chondrocytes may undergo a transition from their inherently stable phenotype to a more transient one characteristic of the chondrocytes in the growth plate 2, 3, 4, 5, 6, 7, 8, 9.

The epiphyseal growth plates are responsible for long bone development (endochondral ossification) and growth, which is secured by growth plate chondrocytes undergoing differentiation, maturation, hypertrophy, and death, resulting in mineralization of the cartilage matrix 10, 11, 12, 13. Transience of growth plate cartilage chondrocytes is thus a crucial attribute. However, this is in sharp contrast with the inherent stability of articular cartilage chondrocytes, in which these dynamic events must be restricted to assure life long articular integrity and joint function. Interlinks between these apparently discordant phenotypes are not fully understood, and whether switching in these behaviors may contribute to the structural demise of articular cartilage in OA joints has not yet been established 13, 14, 15. However, based on the common embryology of cartilage and bone, along with recent evidence supporting distinct origins of growth plate and articular cartilage chondrocytes, it is not surprising that this hypothesis has been controversial 16, 17, 18. Regardless, an exploration of the mechanisms controlling changes that chondrocytes undergo during their transition through the various stages of endochondral ossification may help to decipher those that underlie pathologic ossification in OA.

The STR/Ort mouse is a well‐established, natural model of OA, with disease resembling that in humans. Mice develop articular cartilage lesions on the medial tibial plateau, with subchondral bone thickening and expected degenerative changes in other joint tissues beginning at ∼18 weeks of age, coincident with attainment of skeletal maturity 19, 20, 21, 22. CBA mice, the closest available parental strain, show, in contrast, very low spontaneous OA susceptibility 21, 23. We therefore aimed to establish whether an aberrant deployment of the transient chondrocyte phenotype is observed in STR/Ort mouse joints and whether this can be attributed to modified growth dynamics underpinned by an inherent endochondral growth defect.

## MATERIALS AND METHODS

### Animals

Male STR/Ort mice (bred in‐house) and CBA mice (Charles River) were used in all experiments. All procedures complied with the Animals (Scientific Procedures) Act 1986 and local ethics committee guidelines.

### Meta‐analysis of microarray data

Gene ontology classification, on Affymetrix mouse gene microarray profiling of articular cartilage that we had performed previously 22, was carried out using DAVID (http://david.abcc.ncifcrf.gov/) 24.

### RNA extraction

RNA was extracted from the knee joint articular cartilage of STR/Ort and CBA mice at ages 8–10 weeks, 18–20 weeks, and ≥40 weeks (n = 3 joints per strain per age group), as previously described 22.

### Multiplex quantitative reverse transcriptase–polymerase chain reaction (qRT‐PCR)

A GeXP multiplex qRT‐PCR assay was designed for the following gene targets: *Ank*, *Dmp1*, *Enpp1*, *Mepe*, *Opn (Spp1)*, *Phex*, and *Sost* (see Supplementary Table 1, available on the *Arthritis & Rheumatology* web site at http://onlinelibrary.wiley.com/doi/10.1002/art39508/abstract). Target‐specific reverse transcription was performed as previously described 25, 26, using 50 ng of total RNA.

### Immunohistochemistry

Immunohistochemical analysis was performed on 6‐μm coronal sections using anti‐sclerostin antibody (1:100 dilution; R&D Systems), anti–matrix metalloproteinase (anti–MMP‐13) antibody (1:200 dilution; Abcam), anti‐Col10a1 antibody (1:500 dilution; provided by Professor R. Boot‐Handford, University of Manchester), or anti‐MEPE antibody (1:200 dilution; provided by Professor P. Rowe, University of Kansas Medical Center, Kansas City, Kansas).

### Articular cartilage and growth plate zone analysis.

Multiple toluidine blue–stained coronal sections (n => 6) from the joints of 4 individual mice per strain in each age group were used to measure the width of joint compartments and growth plate zones based on established cell morphology 27.

### Joint imaging by micro–computed tomography (micro‐CT)

Mouse joints were scanned using a laboratory source for 5μ voxels and at a synchrotron for 1μ voxels. The laboratory scans were performed using a SkyScan 1172 x‐ray microtomograph to evaluate cortical and trabecular bone geometry. The synchrotron radiation microtomography was performed at Diamond Light Source on the Diamond‐Manchester Branchline I13‐2 with projections being reconstructed and a procedure developed to characterize each individual bridge and map its location on the tibial joint surface 28, 29, 30 (see Supplementary Methods, available on the *Arthritis & Rheumatology* web site at http://onlinelibrary.wiley.com/doi/10.1002/art39508/abstract).

### Metatarsal organ cultures

Metatarsal bones (day 15 of embryogenesis) were cultured for up to 7 days 31, 32. The total length of the bone through the center of the mineralizing zone and the length of the central mineralization zone were determined using Image J software.

### Sclerostin enzyme‐linked immunosorbent assay (ELISA).

Serum sclerostin levels in CBA and STR/Ort mice at ages 8–10 weeks, 18–20 weeks, and ≥40 weeks (n = 4 for each strain at each age) were measured using a mouse/rat sclerostin ELISA kit (R&D Systems).

### Statistical analysis

Data were analyzed by one‐way analysis of variance, Student's *t*‐test, or a suitable nonparametric test using GraphPad Prism 6 and following normality checks. All data are expressed as the mean ± SEM.

## RESULTS

### Retention of calcified cartilage thickness despite articular cartilage loss and subchondral bone thickening in STR/Ort mice

**Figure 1:**
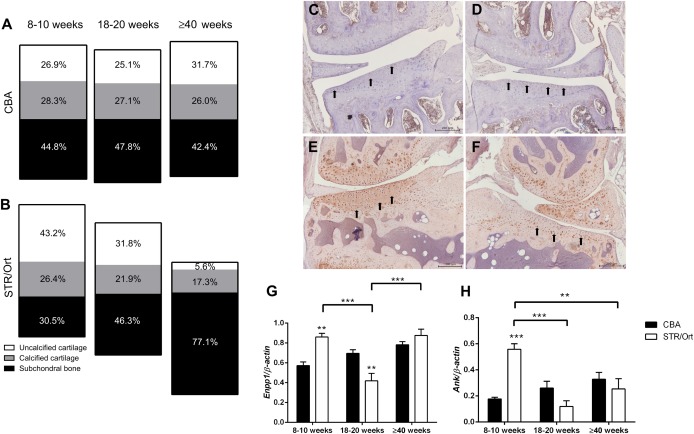
**A** and **B** **,** Thickness of uncalcified cartilage, calcified cartilage, and subchondral bone in the medial tibia of CBA mice (**A**) and STR/Ort mice (**B**) at 8–10 weeks, 18–20 weeks, and ≥40 weeks of age. Ten measurements per section were obtained in >6 sections per mouse (n = 4 mice per age group for each strain). Results are presented as the average percent of the thickness of each zone measured from articular surface to subchondral bone. **C–F** **,** Immunolabeling for matrix metalloproteinase 13 (**C** and **D**) and for type X collagen (**E** and **F**) in the medial tibia (**C** and **E**) and lateral tibia (**D** and **F**) of STR/Ort mice prior to the onset of osteoarthritis. **Arrows** indicate positive staining. Images are representative of results in 3 individual mice. **G** and **H** **,** GeXP multiplex quantitative reverse transcription–polymerase chain reaction analysis of mRNA for *Enpp1* (**G**) and *Ank* (**H**) in the articular cartilage of CBA and STR/Ort mice at 8–10 weeks, 18–20 weeks, and ≥40 weeks of age. Bars show the mean ± SEM (n = 3 joints per sample; n = 3 samples per age group per strain). ∗∗ = *P* < 0.01; ∗∗∗ = *P* < 0.001, versus CBA mice except where indicated otherwise. Color figure can be viewed in the online issue, which is available at http://onlinelibrary.wiley.com/journal/doi/10.1002/art.39508/abstract.

We first sought to determine temporospatial patterns of changing joint architecture in STR/Ort mice. Consistent with previous findings 33, we found that young STR/Ort mice had thicker medial tibial articular cartilage than age‐matched CBA controls (*P* < 0.001). As STR/Ort mice aged, the medial tibial articular cartilage became thinner, with concomitant thickening of subchondral bone, neither of which occurred in CBA mice (*P* < 0.001) (Figures 1A and B). Despite this, there was no change in calcified cartilage thickness in STR/Ort mice (Figure 1B), and further analysis revealed similar changes in the medial femur (see Supplementary Figure 1, available on the *Arthritis & Rheumatology* web site at http://onlinelibrary.wiley.com/doi/10.1002/art39508/abstract). Lateral condyles in STR/Ort mice were unaffected by such age‐related structural modifications. The lateral tibia also exhibited greater articular cartilage thickness, possibly as compensation for altered mechanical loads (see Supplementary Figure 1).

### Transient chondrocyte behaviors in the articular cartilage of STR/Ort mice prior to OA onset

We next examined whether the predisposition to age‐related subchondral bone thickening and loss of articular cartilage in STR/Ort mice with OA was linked to the expression of markers of the transient chondrocyte phenotype. Initially, we used the DAVID functional annotation clustering tool to identify biologic functions enriched within differentially expressed genes in articular cartilage from mice with OA (STR/Ort mice ages >18 weeks) compared to unaffected mice (CBA mice ages 8–40 weeks and STR/Ort mice ages 8–10 weeks) 22. Within the major gene ontology classifications, there was significant up‐regulation of endochondral bone growth (*P* < 0.01) (Table 1 and Supplementary Table 2, available on the *Arthritis & Rheumatology* web site at http://onlinelibrary.wiley.com/doi/10.1002/art39508/abstract). No gene ontology classifications associated with skeletal development and ossification were found to be significantly down‐regulated.

**Table 1:** Gene ontology analysis of genes up‐regulated in osteoarthritic mouse articular cartilage compared to nonosteoarthritic mouse articular cartilage^a^

Term	% change	*P*
Bone development	3.1	1.0 × 10^−5^
Ossification	2.8	2.8 × 10^−5^
Bone mineral formation	1.7	1.6 × 10^−4^
Skeletal system development	4	3.4 × 10^−4^
Cartilage development	1.7	6.10 × 10^−3^

a Genes showing a change of >1.5 fold (n = 491) were analyzed with DAVID.

These endochondral ossification gene ontologies in STR/Ort mouse OA articular cartilage led us to examine protein expression of well‐established chondrocyte hypertrophy markers in OA development. Immunohistochemistry demonstrated positive MMP‐13 labeling in both superficial and deep articular chondrocytes in the joints of STR/Ort mice prior to OA onset (Figures 1C and D). Consistent with previous findings 35, an expected pattern of type X collagen expression was observed in the unaffected condyles of STR/Ort mouse joints, with immunolabeling restricted to hypertrophic chondrocytes (Figure 1F). Intriguingly, type X collagen immunolabeling was observed throughout the medial condylar articular cartilage matrix in 8–10‐week‐old STR/Ort mice, before histologically detectable OA (Figure 1E). Multiplex gene analysis of genes associated with matrix mineralization confirmed significant elevation in *Enpp1* and *Ank* messenger RNA (mRNA) isolated from articular cartilage from 8–10‐week‐old STR/Ort mice, compared to CBA mice (*P* < 0.001) (Figures 1G and H). Interestingly, these increases were diminished upon OA onset (*P* < 0.01 for STR/Ort mice at 18–20 weeks versus age‐matched CBA mice for *Enpp1*) (Figures 1G and H). These findings suggest that OA‐prone regions of the STR/Ort mouse cartilage exhibit an inherent instability defect in articular chondrocytes.

### Accelerated growth, dysfunctional growth plate morphology, and matrix mineralization in STR/Ort mice

**Figure 2:**
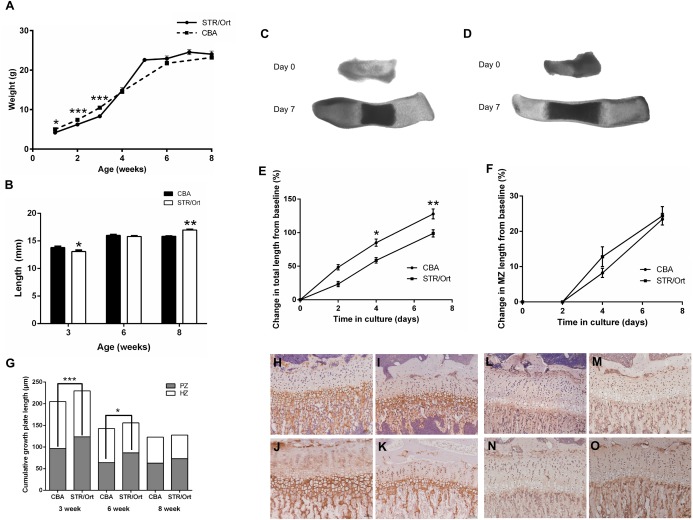
**A,** Weight of CBA and STR/Ort mice from 1 to 8 weeks of age (n = 5 or more mice per group). **B,** Length of the tibia in CBA and STR/Ort mice at 3, 6, and 8 weeks of age (n = 6 mice per group). **C** and **D,** Digital images of metatarsal bones from STR/Ort mice on day 15 of embryogenesis (**C**) and CBA mice (**D**) that were cultured for 7 days and measured. **E** and **F,** Growth rate (**E**) and percent change in mineralization zone (MZ) length (**F**) of metatarsal bones of CBA and STR/Ort mice (n = 8 or more mice per group). **G,** Growth plate zone length in CBA and STR/Ort mice at 3, 6, and 8 weeks of age. Ten measurements per section were obtained in >6 sections per mouse (n = 4 mice per age group for each strain). PZ = proliferative zone; HZ = hypertrophic zone. **H–K,** Immunohistochemical analysis of type X collagen in a 3‐week‐old CBA mouse (**H**), a 6‐week‐old CBA mouse (**I**), a 3‐week‐old STR/Ort mouse (**J**), and a 6‐week‐old STR/Ort mouse (**K**). **L–O,** Immunohistochemical analysis of MMP‐13 in a 3‐week‐old CBA mouse (**L**), a 6‐week‐old CBA mouse (**M**), a 3‐week‐old STR/Ort mouse (**N**), and a 6‐week‐old STR/Ort mouse (**O**). Images are representative of results in 3 individual mice. Values in **A,** **B,** **E,** **F,** and **G** are the mean ± SEM. ∗ = *P* < 0.05; ∗∗ = *P* < 0.01; ∗∗∗ = *P* < 0.001. Original magnification × 10 in **H**–**O**. Color figure can be viewed in the online issue, which is available at http://onlinelibrary.wiley.com/journal/doi/10.1002/art.39508/abstract.

To more fully define this inherent endochondral chondrocyte defect, we examined the growth trajectories of 1–8‐week‐old STR/Ort and CBA mice. We found that STR/Ort mice weigh less than CBA mice (*P* < 0.05) until 4–5 weeks of age, corresponding with the attainment of sexual maturity, when they overtake CBA mice (Figure 2A). Consistent with this finding, analysis of embryonic longitudinal growth and mineralization in vitro revealed aberrant rates of growth and endochondral ossification in STR/Ort mouse metatarsal bones 31, 32 (Figures 2C–F), involving both a slowing of metatarsal growth (Figure 2E) and marked reductions in the mineralized portion of the element (Figure 2F).

Consistent with this accelerated growth phenotype, STR/Ort mice also had shorter tibiae than CBA mice at 3 weeks of age (*P* < 0.05), which seemed to be reversed, since tibia length at 6 weeks of age was not significantly different from that of CBA mice (Figure 2B). This was further supported by comparisons of 8‐week‐old mice, when the tibia was longer in STR/Ort mice than in age‐matched CBA mice (*P* < 0.01) (Figure 2B). Micro‐CT analysis showed significantly enhanced cortical and trabecular parameters in 6‐week‐old STR/Ort mouse tibiae, with higher percent differences in bone volume/total volume (12%; *P* < 0.01), cortical thickness (23%; *P* < 0.05), cortical area (23%; *P* < 0.001), polar moment of inertia (46%; *P* < 0.05), trabecular pattern factor (27%; *P* < 0.05), and structure model index (13%; *P* < 0.05) compared to age‐matched CBA mice (see Supplementary Table 3, available on the *Arthritis & Rheumatology* web site at http://onlinelibrary.wiley.com/doi/10.1002/art39508/abstract). In comparison, there were no significant differences at 3 weeks of age (Supplementary Table 3).

Growth plate zone analysis showed a significantly enlarged proliferating zone of chondrocytes in both 3‐week‐old and 6‐week‐old STR/Ort mice (*P* < 0.001 and *P* < 0.05, respectively) (Figure 2G). This was not apparent in 8‐week‐old STR/Ort mice when compared to age‐matched CBA mice (Figure 2G). Despite the lack of any differences in the size of hypertrophic chondrocyte zones, immunolabeling for type X collagen showed the expected localization in CBA mouse growth plates, limited exclusively to the hypertrophic zone and underlying metaphyseal bone at both 3 weeks and 6 weeks of age (Figures 2H and I). In contrast, STR/Ort mice at both ages showed considerably greater and more widely dispersed type X collagen expression, which extended additionally into the proliferative chondrocyte zone (Figures 2J and K). This disrupted distribution of growth plate zone markers was also evident for MMP‐13 in the growth plates of STR/Ort mice (Figures 2L–O).

### Link between OA in STR/Ort mice and modifications in the MEPE/sclerostin axis

**Figure 3:**
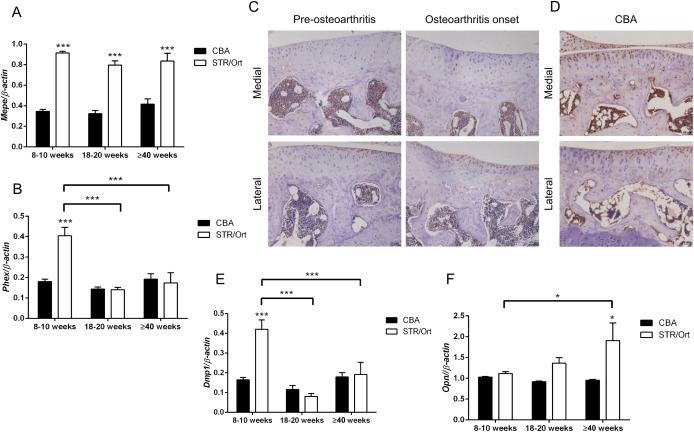
**A** and **B,** GeXP multiplex quantitative reverse transcription–polymerase chain reaction (qRT‐PCR) analysis of mRNA for *Mepe* (**A**) and *Phex* (**B**) in the articular cartilage of CBA and STR/Ort mice at 8–10 weeks, 18–20 weeks, and ≥40 weeks of age (n = 3 joints per sample; n = 3 samples per age group per strain). **C,** Immunohistochemical analysis of MEPE in the medial (affected) and lateral (unaffected) tibial condyles of STR/Ort mice prior to and at the onset of osteoarthritis. **D,** Immunohistochemical analysis of MEPE in the tibial condyles of a CBA mouse. Images are representative of results in 3 individual mice. **E** and **F,** GeXP multiplex qRT‐PCR analysis of mRNA for *Dmp1* (**E**) and *Opn* (**F**) in the articular cartilage of CBA and STR/Ort mice at 8–10 weeks, 18–20 weeks, and ≥40 weeks of age (n = 3 joints per sample; n = 3 samples per age group per strain). Bars in **A,** **B,** **E,** and **F** show the mean ± SEM. ∗ = *P* < 0.05; ∗∗∗ = *P* < 0.001, versus CBA mice except where indicated otherwise. Color figure can be viewed in the online issue, which is available at http://onlinelibrary.wiley.com/journal/doi/10.1002/art.39508/abstract.

Molecular mechanisms controlling endochondral ossification may help identify those involved in OA. We have previously shown MEPE, a member of the small integrin‐binding ligand *N*‐linked glycoprotein (SIBLING) family, to be a negative regulator of growth plate chondrocyte matrix mineralization 31. Examination of MEPE expression by multiplex analysis showed significantly higher levels of *Mepe* mRNA in STR/Ort mouse articular cartilage than CBA mouse articular cartilage (*P* < 0.001) (Figure 3A). Immunolabeling for MEPE showed differential expression across the tibia of the STR/Ort mouse joints, with a distinct lack of positive MEPE protein labeling in the medial (affected) aspects both prior to and during OA progression (Figure 3C). This is in contrast to the lateral (unaffected) aspect of the STR/Ort mouse joints and throughout all aspects of CBA mouse joints, where labeling for MEPE was observed throughout the depth of the articular cartilage tissue (Figures 3C and D).

Assessment of the MEPE regulator *Phex* revealed elevated mRNA levels in articular cartilage from 8–10‐week‐old STR/Ort mice compared to age‐matched CBA mice (*P* < 0.001) (Figure 3B). In STR/Ort mice, this expression significantly decreased with OA onset (*P* < 0.001 for STR/Ort mice at 18–20 weeks versus STR/Ort mice at 8–10 weeks) (Figure 3B). Identical age‐related expression patterns were found for *Dmp1* mRNA, another SIBLING family member (*P* < 0.001) (Figure 3E). Analysis of mRNA levels of osteopontin (*Opn*), a SIBLING family member with shared roles in biomineralization, showed significantly higher levels in aged STR/Ort mice compared to young STR/Ort mice (*P* < 0.05) and age‐matched CBA mice (*P* < 0.05), resembling patterns of *Mepe* expression (Figure 3F). Taken together, these findings suggest a regulatory role for the SIBLING family of proteins in OA development in these mice.

**Figure 4:**
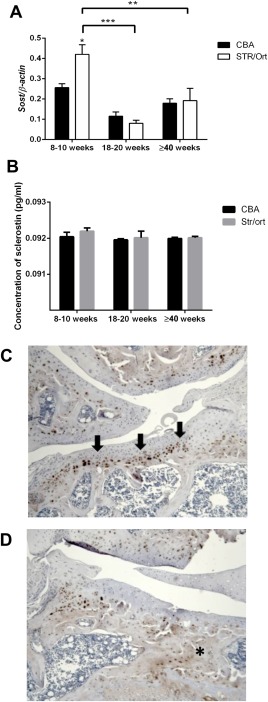
**A**, GeXP multiplex quantitative polymerase chain reaction analysis of mRNA for *Sost* in the articular cartilage of CBA and STR/Ort mice at 8–10 weeks, 18–20 weeks, and ≥40 weeks of age (n = 3 joints per sample; n = 3 samples per age group per strain). **B,** Serum sclerostin levels in CBA and STR/Ort mice at 8–10 weeks, 18–20 weeks, and ≥40 weeks of age (n = 4 mice per age group for each strain). Bars in **A** and **B** show the mean ± SEM. **C** and **D,** Immunohistochemical analysis of sclerostin in the lateral (unaffected) tibial condyles (**C**) and medial (affected) tibial condyles (**D**) in STR/Ort mice at the onset of osteoarthritis. **Arrows** in **C** indicate sclerostin immunolabeling. **Asterisk** in **D** indicates subchondral bone thickening. Images are representative of results in 3 individual mice. Color figure can be viewed in the online issue, which is available at http://onlinelibrary.wiley.com/journal/doi/10.1002/art.39508/abstract.

We next sought to examine the temporal expression of another important regulator of MEPE expression, the Wnt signaling inhibitor sclerostin (*Sost*) 36. Our analyses showed greater levels of *Sost* mRNA in articular cartilage from 8–10‐week‐old STR/Ort mice than that from age‐matched CBA mice (*P* < 0.05) (Figure 4A), with levels significantly decreasing with OA onset (*P* < 0.01 for STR/Ort mice at 8–10 weeks versus STR/Ort mice at 18–20 weeks) (Figure 4A). Despite this, no differences in circulating serum sclerostin concentrations were observed in these mice at any age (Figure 4B), indicating solely local effects. Consistent with this finding, sclerostin immunolabeling showed a clear enrichment in cells at the osteochondral interface in unaffected regions of STR/Ort mouse joints (Figure 4C). In contrast, STR/Ort mice with OA showed suppression of positive sclerostin labeling of regions of subchondral bone thickening underlying those with compromised articular cartilage integrity (Figure 4D).

### Link between premature growth plate closure in STR/Ort mice and OA development

**Figure 5:**
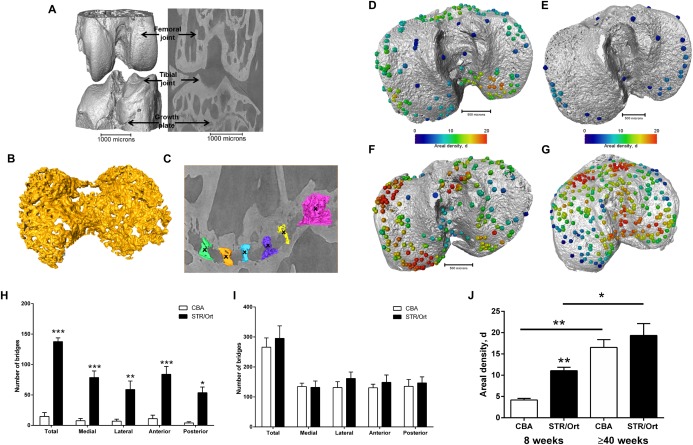
Development of a 3‐dimensional quantification method for growth plate bridging. **A,** Three‐dimensional representation of an entire joint from an STR/Ort mouse at ≥40 weeks of age. **B,** Three‐dimensional representation of the growth plate cartilage (yellow) underneath the tibial joint surface (shown in gray in **A**). **C,** Three‐dimensional representation of bridges crossing the growth plate underneath the tibial joint. Crosses indicate bony bridges identified by an observer. **D–G,** Location and areal density of bridges across the growth plate projected on the tibial joint surface in an STR/Ort mouse at 8 weeks of age (**D**), a CBA mouse at 8 weeks of age (**E**), an STR/Ort mouse at ≥40 weeks of age (**F**), and a CBA mouse at ≥40 weeks of age (**G**). **H** and **I,** Number of bridges per tibia in CBA and STR/Ort mice at 8 weeks of age (**H**) and ≥40 weeks of age (**I**). The lateral and medial segments and anterior and posterior segments were split in order to examine whether bridging is balanced during fusion. **J,** Areal density (d) of bridges, defined as the number of bridges per 256 μm × 256 μm window. Bars in **H–J** show the mean ± SEM (n = 3 mice per group). ∗ = *P* < 0.05; ∗∗ = *P* < 0.01; ∗∗∗ = *P* < 0.001, versus CBA mice except where indicated otherwise.

To directly test whether longitudinal growth, growth plate fusion, and OA exhibit interrelationships in STR/Ort mice, we developed a novel protocol for quantifying bony bridges formed across the entire murine tibia epiphysis during growth plate fusion (see Supplementary Methods, available on the *Arthritis & Rheumatology* web site at http://onlinelibrary.wiley.com/doi/10.1002/art39508/abstract) (Figures 5A–C). Applying this novel method to examine growth plate closure in STR/Ort mice and CBA mice at 8 weeks of age and ≥40 weeks of age revealed a dramatically (10‐fold) greater total number of bridges in 8‐week‐old STR/Ort mice (mean ± SEM 137 ± 10) than in CBA mice (mean ± SEM 14 ± 10) (*P* < 0.001) (Figures 5D, E, and H) (see Supplementary Figure 2, available on the *Arthritis & Rheumatology* web site at http://onlinelibrary.wiley.com/doi/10.1002/art39508/abstract). This enriched growth plate bridging was apparent in all aspects of STR/Ort mouse tibiae (*P* < 0.05) (Figure 5H). Although still evident in aged STR/Ort mice (≥40 weeks), the enriched bone bridging was much less pronounced (mean ± SEM 295 ± 72 in STR/Ort mice and 266 ± 53 in CBA mice) (Figures 5F, G, and I and Supplementary Figure 2). Mean areal bridge densities were also greater in STR/Ort mice at both ages (*P* < 0.01) (Figure 5J). These intriguing data reveal an accelerated cartilage–bone transition in the growth plate which, taken together with our findings described above, support the notion of an inherent endochondral defect in both the articular and growth plate cartilage in STR/Ort mice.

## DISCUSSION

Our data reveal changes in the articular cartilage of STR/Ort mouse knee joints consistent with an aberrant deployment of endochondral processes. This is associated with inherent longitudinal growth modifications, disrupted growth plate morphology, premature growth plate fusion, and aberrant bone formation and matrix mineralization prior to OA onset. These data indicate that, at least in the spontaneous human‐like OA seen in STR/Ort mice, growth‐related endochondral ossification abnormalities may forecast mechanisms of OA development in articular cartilage.

There are certainly some intriguing previously published data on the expression of endochondral ossification markers that support this notion 14. Type X collagen is a marker of chondrocyte hypertrophy that is usually found in the growth plate and is unique to the calcified cartilage in normal joints 36. Expression of type X collagen mRNA transcripts, as examined by in situ hybridization, has, however, been observed throughout articular cartilage in both young STR/Ort mice (at 9 weeks of age) and older STR/Ort mice (at ≥41 weeks of age) 35. This is the first study to provide evidence of associated type X collagen protein expression in these mice. Consistent with our findings, an additional marker of chondrocyte hypertrophy, MMP‐13, has been detected in the calcified cartilage chondrocytes of STR/Ort mice at both young and old ages, at levels greater than those in age‐matched CBA mice 37. Similarly, higher expression levels of several other MMPs (MMP‐2, MMP‐3, MMP‐7, MMP‐9, and membrane type 1 MMP) were observed in the tibial articular chondrocytes of the STR/Ort mouse 37. Indeed, many of these MMPs were also shown to be significantly increased in our previous microarray study, further highlighting their likely role as key players in cartilage degradation in OA 22.

The STR/Ort mouse growth plate has remained relatively unexamined with, to our knowledge, only one published report describing phenotypic changes associated with aging. Chambers et al 35 describe type X collagen mRNA expression localized to hypertrophic chondrocytes, as expected, in both young CBA mice and young STR/Ort mice. However, in the older mice, no expression of type X collagen mRNA was observed, despite the preservation of type II collagen mRNA throughout the depth of the thinned growth plate cartilage 35. The results of the present study indicate aberrant expression of type X collagen and MMP‐13 additionally in the growth plate of young STR/Ort mice. STR/Ort mice also display an increased zone of proliferative chondrocytes, based on well‐established cell morphologic features 27. These results may seem counterintuitive, but they highlight the fact that there is clearly an inherent endochondral defect in STR/Ort mice, which may also precipitate OA pathogenesis.

Molecular mechanisms controlling endochondral ossification may help identify those involved in OA. Effective control of the Wnt signaling pathway is certainly proving critical in regulating both the extent of OA joint pathology 38 and growth plate chondrocyte behavior, and the data in the present study corroborate this.

Genetic and microarray analyses have been performed in STR/Ort mice in order to better elucidate the etiology of their OA 39, 40, 41, 42. Jaeger and colleagues identified a quantitative trait locus (QTL) associated with articular cartilage degeneration on chromosome 8 of the STR/Ort mouse 39. This, however, was not corroborated in a more recent QTL analysis in which STR/Ort mice were backcrossed with the C57BL/6N strain 43. This QTL was therefore suggested to be a recessive trait among the polygenetic factors in OA in STR/Ort mice 19, 43. Instead, the authors identified a QTL for the OA phenotype that is mapped to chromosome 4 43. Chromosome 8 was, however, revisited, and fine‐mapping of the OA QTL in a more recent study revealed Wnt‐related genes associated with altered chondrogenesis, including dickkopf 4 (*Dkk4*), secreted Frizzled‐related protein 1 (*Sfrp1*), and fibroblast growth factor 1 (*Fgfr1*) 38, 42. While a number of genes, including Wnt‐related genes, have been implicated in OA by association studies in human populations, there is a distinct lack of functional data to support a causative link between these associated genes and OA.

Pasold et al attempted to find such a link and identified 23 polymorphic changes in the *Sfrp1* gene in STR/Ort mice in comparison to C57BL/6 mice 42. Further immunohistochemical studies demonstrated that the expression of secreted Frizzled‐related protein 1 was reduced in articular chondrocytes from young STR/Ort mice, and this finding was confirmed by in vitro analysis of STR/Ort mesenchymal stem cells 42. In the present study, our meta‐analysis of previous STR/Ort microarray data did not reveal any significant changes in the gene expression of *Dkk4, Sfrp1,* or *Fgfr1*. Instead, we found evidence of the role of the Wnt inhibitor sclerostin in OA development in STR/Ort mice. This is consistent with the findings of other studies that have shown expression of sclerostin in the articular cartilage of various species, including mice and sheep 44, 45. While we observed no differences in serum sclerostin levels in STR/Ort mice, in humans an inverse relationship with radiographic knee OA severity has been observed, therefore implicating sclerostin as a potential biochemical marker 46.

This does not mean that the role of sclerostin in OA is not controversial. Recent studies examining OA in aged sclerostin‐deficient mice and in rats treated with sclerostin‐neutralizing antibodies following surgical induction 45 concluded that genetic ablation of sclerostin does not alter spontaneous age‐dependent murine OA development, nor does pharmacologic inhibition of sclerostin in a surgical model of OA 45. This therefore highlights the growing need for further investigation into the precise role of sclerostin in this debilitating disease. This may come from examining the role of its downstream pathways; in this study we were interested in identifying the role of one such novel pathway involving the SIBLING protein MEPE 36. MEPE has been shown to be a critical regulator of osteoblast and chondrocyte matrix mineralization 12, 31, 47, 48. The similarity in differential patterns of MEPE and sclerostin expression that we observed in STR/Ort mice implies a novel mechanism by which sclerostin may function in OA. Alterations in this pathway support the case for abnormal Wnt/β‐catenin signaling, as has been demonstrated in many studies of OA, including in the STR/Ort mouse 42.

In endochondral growth, the Wnt pathway is known to play an intricate and yet critical role, with cartilage‐specific β‐catenin–deficient mice lacking typical growth plate zones and exhibiting delayed endochondral ossification 49. It has been shown that appropriate control of Wnt signaling in the growth plate is essential in regulating proliferation, alignment, differentiation, hypertrophy, and replacement of calcified matrix with bone 38. During chondrogenesis, Wnt signaling is thought to influence the cell–cell and cell–extracellular matrix interactions upon which this fundamental process depends. Various Wnt factors, including Wnt‐3a, Wnt‐6, Wnt‐7a, and many more, have been implicated as inhibitors of chondrogenesis, while a similar number, including Wnt‐5a and Wnt‐5b, have stimulatory roles 38.

Similar ambiguity applies to the role of Wnt signaling in chondrocyte hypertrophic differentiation 38. In particular, it has been shown that Wnt signaling regulates the parathyroid hormone–related protein (PTHrP), Indian hedgehog (IHH), and transforming growth factor β (TGFβ) feedback loop 49. Chondrocytes undergoing hypertrophy secrete IHH, which acts upon the proliferating chondrocytes to maintain their proliferative state and to restrict hypertrophy. IHH also stimulates TGFβ production, which in turn up‐regulates PTHrP. This acts on the prehypertrophic chondrocytes to prevent their further differentiation and thus IHH production 50. This pathway has also been implicated in OA, with increased IHH expression reported in OA cartilage and selective IHH deletion protecting against surgical OA progression 51, 52. While this pathway was not examined in the present study, it would be interesting to investigate whether sclerostin/MEPE regulates the PTHrP/IHH pathway in STR/Ort mice and whether this contributes to their OA pathology. Our data do, however, strengthen evidence of the relationship between molecular dysregulation of the Wnt/β‐catenin pathway and endochondral growth defects.

Whether the results presented here are unique to OA in the STR/Ort mouse or are characteristic of OA in general is an interesting consideration and one that should be deliberated upon, as OA is being more widely accepted as a clinicopathologic syndrome with multiple etiologies. An elegant and comprehensive microarray study by Bateman and colleagues 53 in which gene expression profiling was performed in cartilage from wild‐type mice with surgically induced OA (destabilization of the medial meniscus [DMM]) at 1, 2, and 6 weeks after surgery details the full list of differentially expressed genes between mice with DMM OA and sham‐operated mice. Bateman et al found that levels of the marker of hypertrophy MMP‐13 were unchanged in mice with DMM OA compared to sham‐operated mice at all stages after surgery 53. This contrasts with our findings in STR/Ort mice, in which elevated MMP‐13 expression levels were found prior to and during OA. Consistent with our data, Bateman et al found *Col10a1* to be significantly increased in mice with DMM OA compared to sham‐operated mice at 1 and 2 weeks after surgery and the matrix mineralization regulators *Enpp1* and *Ank* to be increased at all time points after surgery 53. The differential expression of these matrix and mineralization markers at early time points after surgery suggests their involvement in the initial OA processes in the DMM model. This is consistent with our data, which show similar changes prior to OA development in STR/Ort mice. Taken together, these findings suggest a point of integration with these endochondral pathways at which the different OA subtypes, surgical (DMM) and natural (STR/Ort), may converge.

In this report, we highlight the MEPE/sclerostin pathway as a potential pathway for future investigation in OA research. Our data show differential expression of MEPE and sclerostin in the STR/Ort mouse, along with the MEPE regulator PHEX and other members of the SIBLING family of proteins, DMP1 and osteopontin. In the DMM model, none of these genes of interest were dysregulated 53. Therefore, this subset of genes is specific to STR/Ort mice with OA, and our identification of this molecular phenotype not only will aid understanding of this diverse human condition, but also suggests that we may be able to identify specific gene signatures within particular at‐risk human patients.

Our report of an inherent endochondral defect in STR/Ort mice is further strengthened by our data acquired using synchrotron x‐ray computed microtomography that showed premature growth plate closure in STR/Ort mice. This novel method for 3‐dimensional quantification of bony bridging will no doubt advance understanding of growth plate closure mechanisms, and our unique data revealing the complex internal topographies of the growth plate cartilage layer in CBA and STR/Ort mice (Figures 5B and D) may also yield more insights into the micro‐mechanical environment of the cells in the growth plate 54.

With this method we have demonstrated that OA‐prone STR/Ort mice and healthy CBA mice both display overt bone bridges prior to growth cessation. More specifically, spatial localization of these bridges has shown greater clustering in STR/Ort mice, suggesting that their formation is driven by local factors, likely altered mechanical loading. The idea that OA in STR/Ort mice is driven by loading has certainly been supported by the findings of previous studies suggesting an association with medial patellar dislocation 55 and by those showing accelerated OA following mechanical joint loading 33. There have also been direct links made between growth plate function and mechanical loads, but whether this extends to the regulation of growth plate closure has yet to be explored 56. Regardless, it remains clear that these studies have, for the first time, shown that early growth plate closure is indicative of modified growth trajectory in STR/Ort mice and that growth ceases sooner in the lifetime of these OA‐prone mice than in the closely related parental CBA strain, which shows healthy joint aging.

These data may also be interpreted as evidence that the OA in STR/Ort mice is secondary to a chondrodysplasia. Data indicating internal rotation of the tibia of STR/Ort mice may offer support for this notion 57. However, the linked deployment of transient chondrocyte behaviors in the articular cartilage prior to overt OA development that we observed suggests, instead, that this reflects an inherent chondrocyte defect. Nonetheless, the likelihood that such relationships with modified growth trajectories are also prevalent in human OA is yet to be defined, and longitudinal studies examining associations between growth plate dynamics and OA development in human patients would be particularly informative in our understanding of OA in general.

Despite the predictable disease development in STR/Ort mice, with characteristics resembling those seen in human OA, including osteophytes, subchondral bone sclerosis, and synovial hyperplasia 19, 20, 58, the insights into the etiology of OA provided by our data from this mouse model are limited. For instance, the etiology of OA in STR/Ort mice is not yet known, despite extensive genetic and microarray analyses 39, 40, 41. As such, there are potential factors that may confound interpretation of our data, and it is vital to highlight that our findings define only the distinct pathophysiologic mechanisms important in this subset of OA. In addition, the molecular phenotype we describe is unmodified in CBA mice, suggesting that the phenotype is disease specific, and indeed specific to OA in STR/Ort mice, as opposed to any result of aging. It is still important, however, to consider the molecular phenotype of this particular OA, since it is becoming more widely accepted that generalization of the OA disease in our pursuit of a disease‐modifying treatment is somewhat distracting and flawed 59.

In conclusion, our findings show that aberrant deployment of transient chondrocyte behaviors, consistent with reinitiation of endochondral processes, occurs in the joints of STR/Ort mice, which spontaneously develop OA. Articular cartilage transcription profiles, labeling for endochondral markers, and age‐related growth plate dynamics, both in vivo and in vitro, support the notion that OA in STR/Ort mice is related to an inherent endochondral growth defect that is subject to regulation by the MEPE/sclerostin axis. Further investigation will determine whether this is an underlying mechanism in some or all forms of pathologic ossification in OA.

## AUTHOR CONTRIBUTIONS

All authors were involved in drafting the article or revising it critically for important intellectual content, and all authors approved the final version to be published. Dr. Pitsillides had full access to all of the data in the study and takes responsibility for the integrity of the data and the accuracy of the data analysis.

**Study conception and design.** Staines, Madi, Mirczuk, Burleigh, Poulet, Hopkinson, Bodey, Fowkes, Farquharson, Lee, Pitsillides.

**Acquisition of data.** Staines, Madi, Mirczuk, Parker, Burleigh, Poulet, Hopkinson, Bodey, Fowkes, Farquharson.

**Analysis and interpretation of data.** Staines, Madi, Parker, Lee, Pitsillides.

## Supporting information


**Supplementary Figure 1.** Measurements of uncalcified cartilage (white), calcified cartilage (grey) and subchondral bone (black) in the **(A)** lateral tibia **(B)** lateral femur **(C)** medial femur of CBA and STR/Ort mice at 8‐10 weeks (prior to OA), 18‐20 weeks (onset of OA), and 40+ weeks (severe OA). Measurements were taken of multiple sections (n>6) from the joints of 4 individual mice (at each age) at 10 different points across the joint.

**Supplementary Figure 2.** Location and areal density of bridges across the growth plate projected on the tibial joint surface: CBA 8 weeks, STR/Ort 8 weeks, CBA 40+ weeks, STR/Ort 40+ weeks. Images shown are for each individual mouse analysed (n=3/age and strain).

**Supplementary Figure 3.** Development of a 3D quantification method for growth plate bridging. 3D representation of the growth plate cartilage (yellow) underneath the tibial joint surface (grey) **(A & B)** STR/Ort 40 weeks (Tb.Th = 67 ± 24 µm, S/V = 0.101, SMI = 2), **(C & D)** CBA 40 weeks (Tb.Th = 49 ± 17 µm, S/V = 0.146, SMI = 1.79). Tb.Th, S/V and SMI refer to the average cartilage thickness, surface‐area‐to‐volume‐ratio and structural model index. The SMI determines the plate‐ or rode‐like geometry of the growth plate cartilage structure. An ideal plate, cylinder and sphere have SMI values of 0, 3 and 4, respectively.

**Supplementary Table 1**: Primer sequences used for multiplex RT‐qPCR
**Supplementary Table 2**: Genes associated with the significantly affected gene ontologies detailed in Supplementary Table 3
**Supplementary Table 3**: MicroCT analysis of cortical and trabecular parameters in 3‐ & 6‐ week tibia ± SEM (STR/Ort and CBA mice, n = 3)

Supplementary Information
