# Supplementary figures and images for: Endochondral Growth Defect and Deployment of Transient Chondrocyte Behaviors Underlie Osteoarthritis Onset in a Natural Murine Model
Source: Arthritis Rheumatol. 2016 Mar 28;68(4):880–91. doi: 10.1002/art.39508 (PMC4832379; doi:10.1002/art.39508)

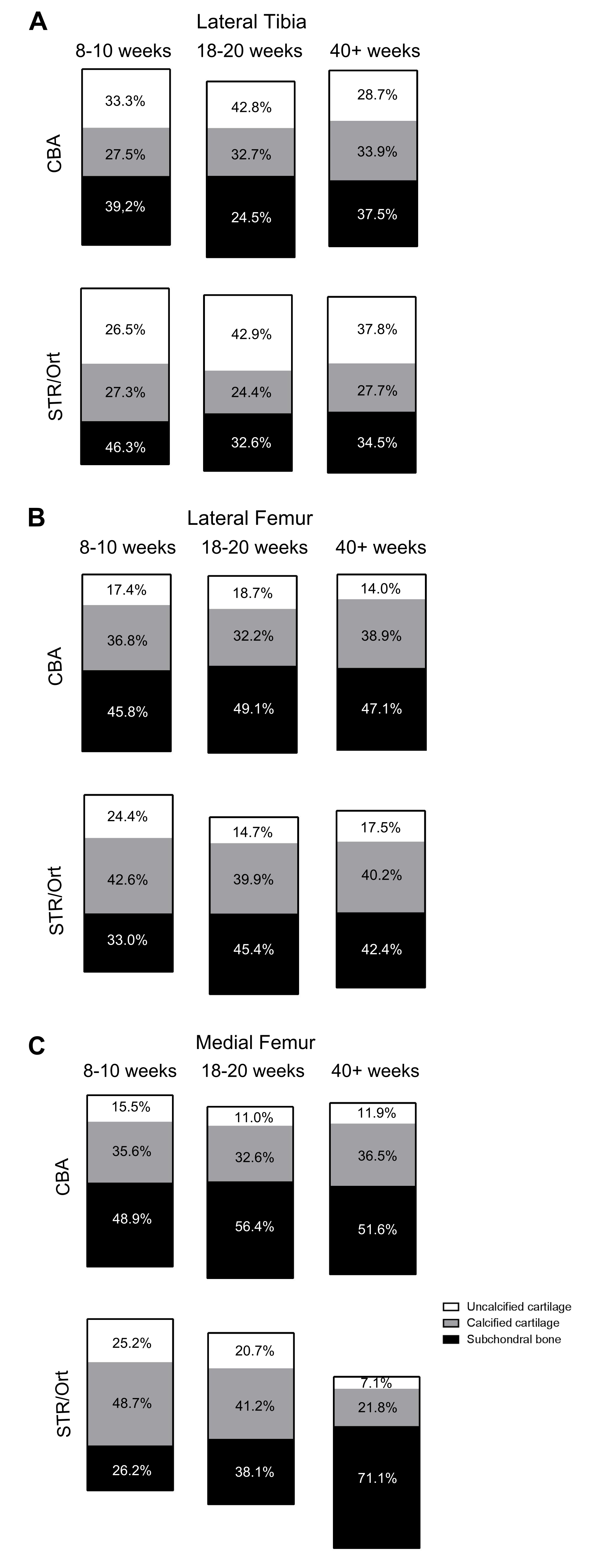

Supplement: Supplementary file 1 — Supplementary Figure 1. Measurements of uncalcified cartilage (white), calcified cartilage (grey) and subchondral bone (black) in the (A) lateral tibia (B) lateral femur (C) medial femur of CBA and STR/Ort mice at 8‐10 weeks (prior to OA), 18‐20 weeks (onset of OA), and 40+ weeks (severe OA). Measurements were taken of multiple sections (n>6) from the joints of 4 individual mice (at each age) at 10 different points across the joint. [file ART-68-880-s001.tif]

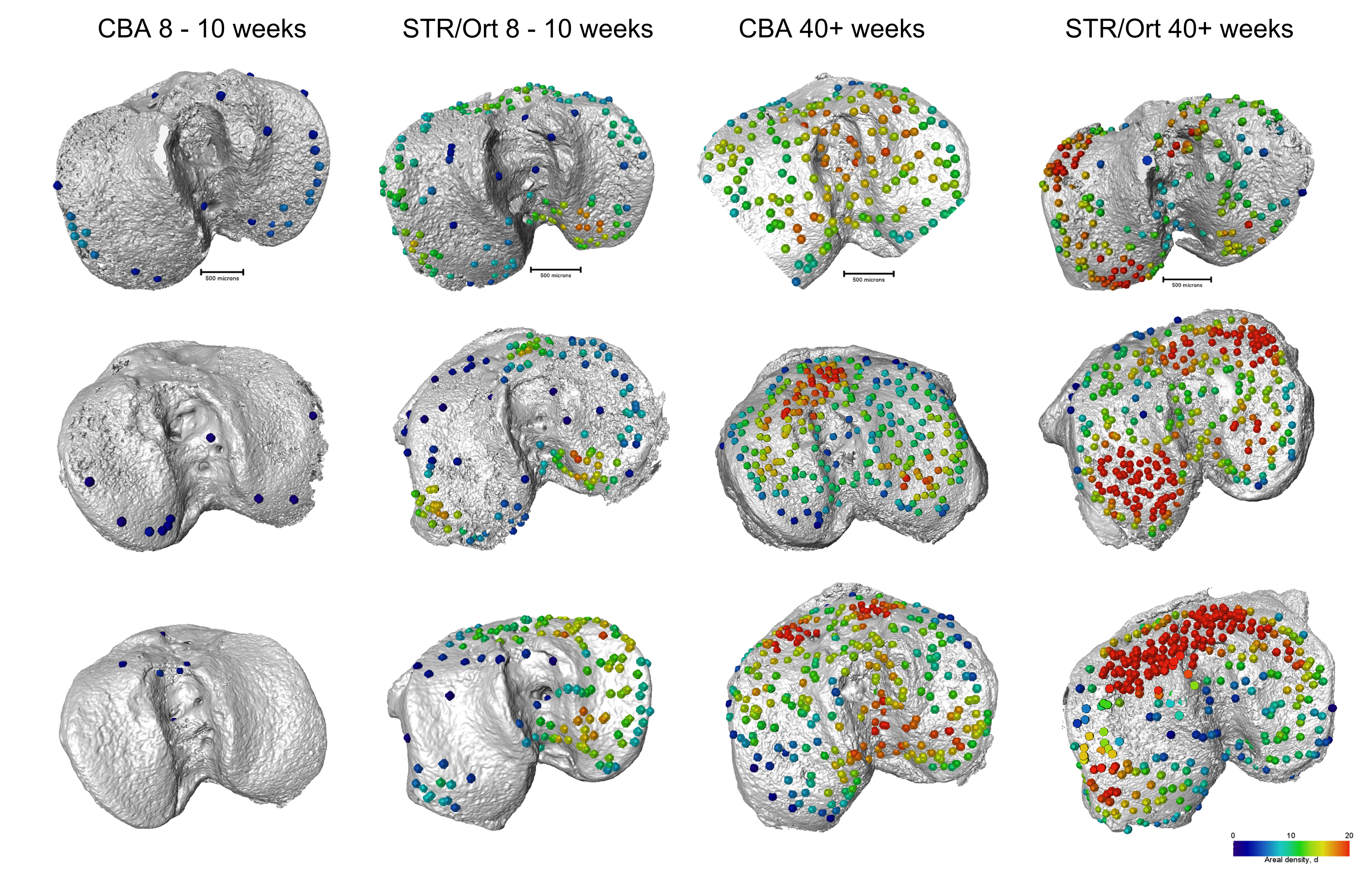

Supplement: Supplementary file 2 — Supplementary Figure 2. Location and areal density of bridges across the growth plate projected on the tibial joint surface: CBA 8 weeks, STR/Ort 8 weeks, CBA 40+ weeks, STR/Ort 40+ weeks. Images shown are for each individual mouse analysed (n=3/age and strain). [file ART-68-880-s002.tif]

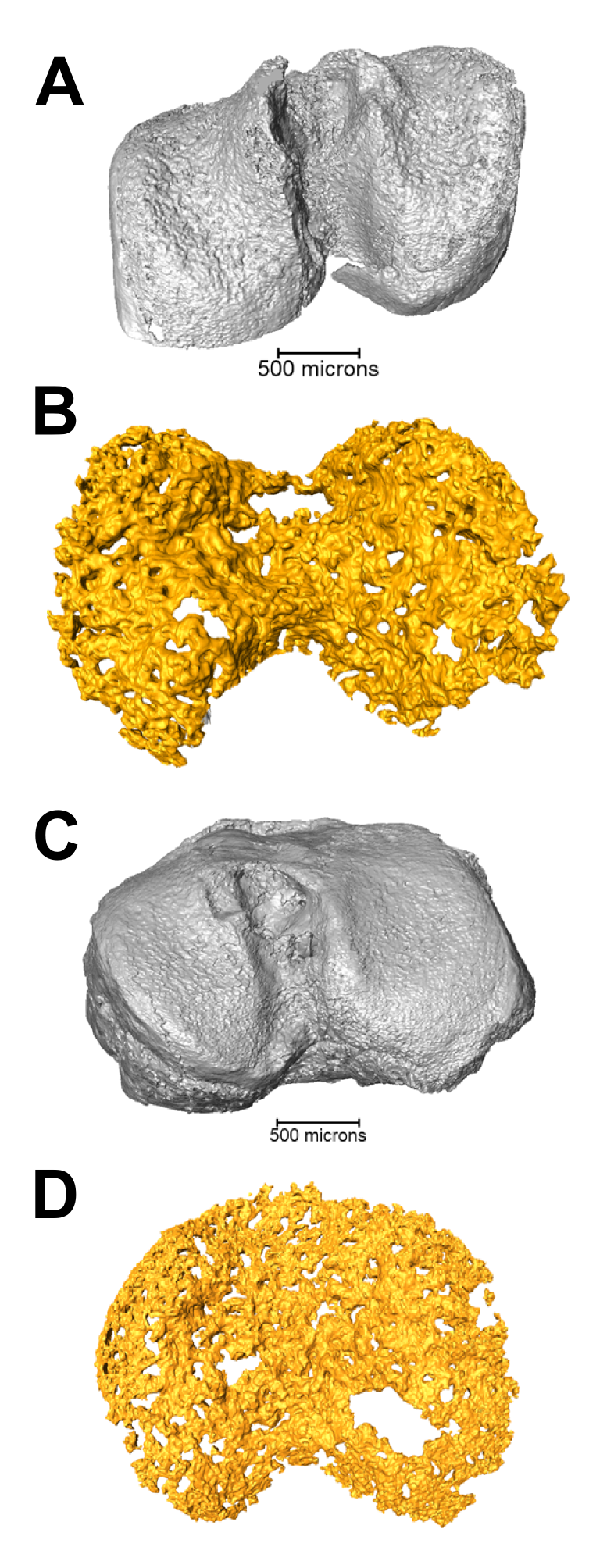

Supplement: Supplementary file 3 — Supplementary Figure 3. Development of a 3D quantification method for growth plate bridging. 3D representation of the growth plate cartilage (yellow) underneath the tibial joint surface (grey) (A & B) STR/Ort 40 weeks (Tb.Th = 67 ± 24 µm, S/V = 0.101, SMI = 2), (C & D) CBA 40 weeks (Tb.Th = 49 ± 17 µm, S/V = 0.146, SMI = 1.79). Tb.Th, S/V and SMI refer to the average cartilage thickness, surface‐area‐to‐volume‐ratio and structural model index. The SMI determines the plate‐ or rode‐like geometry of the growth plate cartilage structure. An ideal plate, cylinder and sphere have SMI values of 0, 3 and 4, respectively. [file ART-68-880-s003.tif]
