# Supplementary material for: Endochondral Growth Defect and Deployment of Transient Chondrocyte Behaviors Underlie Osteoarthritis Onset in a Natural Murine Model
Source: Arthritis Rheumatol. 2016 Mar 28;68(4):880–91. doi: 10.1002/art.39508 (PMC4832379; doi:10.1002/art.39508)
Supplement: Supplementary file 4 — Supplementary Table 1: Primer sequences used for multiplex RT‐qPCR Supplementary Table 2: Genes associated with the significantly affected gene ontologies detailed in Supplementary Table 3 Supplementary Table 3: MicroCT analysis of cortical and trabecular parameters in 3‐ & 6‐ week tibia ± SEM (STR/Ort and CBA mice, n = 3) [file ART-68-880-s004.doc]

**Supplementary Table 1:** Primer sequences used for multiplex RT-qPCR

| **Gene** | **Forward sequence** | **Reverse sequence** |
| --- | --- | --- |
| *Ank* | AGGTGACACTATAGAATATCTTCCACACCCTGATAGCC | GTACGACTCACTATAGGGATGGCATCCATAAAAGGGAAG |
| *Dmp1* | AGGTGACACTATAGAATAGAGTACCAGGAGCGATCGAG | GTACGACTCACTATAGGGACTCAGAGACGTGGGACCTTC |
| *Enpp1* | AGGTGACACTATAGAATATCTACCTGGACCCTCAGTGG | GTACGACTCACTATAGGGAGGCAGGTCCATAGCCAATAA |
| *Mepe* | AGGTGACACTATAGAATAAGGGAAGGAAACCAGGAGAA | GTACGACTCACTATAGGGAGGTGTGTTTGGTGTGTTTGC |
| *Spp1* | AGGTGACACTATAGAATATCTGATGAGACCGTCACTGC | GTACGACTCACTATAGGGAAGGTCCTCATCTGTGGCATC |
| *Phex* | AGGTGACACTATAGAATACAGGCATCACATTCACCAAC | GTACGACTCACTATAGGGAATGGCACCATTGACCCTAAA |
| *Sost* | AGGTGACACTATAGAATAGCCGGACCTATACAGGACAA | GTACGACTCACTATAGGGACCAACATCACACTCAGTGGC |
| *ActB* | AGGTGACACTATAGAATATACAGCTTCACCACCACAGC | GTACGACTCACTATAGGGATCTCCAGGGAGGAAGAGGAT |
| *Rpl19* | AGGTGACACTATAGAATATGGATCCCAATGAGACCAAT | GTACGACTCACTATAGGGAGGAATGGACAGTCACAGGCT |
| *Kanamycin* | AGGTGACACTATAGAATAATCATCAGCATTGCATTCGATTCCTGTTTG | GTACGACTCACTATAGGGAATTCCGACTCGTCCAACATC |

**Supplementary Table 2:** Genes associated with the significantly affected gene ontologies detailed in Supplementary Table 3

| **Affymetrix Exon Gene ID** | **Gene Name** | **Gene ontology** | **Fold change** |
| --- | --- | --- | --- |
| 10382328 | SRY-box containing gene 9 | Bone development, Skeletal system development, Cartilage development | 1.66 |
| 10380419 | collagen, type I, alpha 1 | Bone development, Ossification, Skeletal system development, Cartilage development | 1.64 |
| 10362201 | connective tissue growth factor | Bone development, Skeletal system development, Cartilage development | 1.90 |
| 10523693 | dentin matrix protein 1 | Bone development, Ossification, Bone mineral formation, Skeletal system development | 1.71 |
| 10548879 | matrix Gla protein | Bone development, Ossification, Bone mineral formation, Skeletal system development, Cartilage development | 1.71 |
| 10583044 | matrix metallopeptidase 13 | Bone development, Ossification, Bone mineral formation, Skeletal system development, Cartilage development | 1.87 |
| 10415052 | matrix metallopeptidase 14 (membrane-inserted) | Bone development, Ossification, Skeletal system development | 1.56 |
| 10573924 | matrix metallopeptidase 2 | Bone development, Ossification, Skeletal system development | 1.65 |
| 10350516 | prostaglandin-endoperoxide synthase 2 | Bone development, Ossification, Bone mineral formation, Skeletal system development | 2.17 |
| 10523717 | secreted phosphoprotein 1 | Bone development, Ossification, Bone mineral formation, Skeletal system development | 1.79 |
| 10523701 | similar to Integrin binding sialoprotein; integrin binding sialoprotein | Bone development, Ossification, Bone mineral formation, Skeletal system development | 1.65 |
| 10368289 | ectonucleotide pyrophosphatase/  phosphodiesterase 1 | Bone mineral formation | 1.59 |
| 10554249 | aggrecan | Skeletal system development, Cartilage development | 2.29 |
| 10593225 | zinc finger and BTB domain containing 16 | Skeletal system development | 1.95 |
| 10410248, 10410237 | zinc finger protein 640 | Skeletal system development | 1.87 |

**Supplementary Table 3**: MicroCT analysis of cortical and trabecular parameters in 3- & 6- week tibia ± SEM (STR/Ort and CBA mice, n = 3)

| **Age** | **Genotype** | **% Cortical BV/TV** | **Cortical thickness (μm)** | **Cortical area (mm^2^)** | **Closed porosity (%)** | **Polar inertia of moment (mm^4^)** | **% Trab.**  **BV/TV** | **Trab. thickness (μm)** | **Trab. Number (TbN/μm)** | **Trab. Separation (μm)** | **Trab. pattern factor** | **SMI** |
| --- | --- | --- | --- | --- | --- | --- | --- | --- | --- | --- | --- | --- |
| 3 week | CBA | 44.8 ± 7.1 | 37.4 ± 7.2 | 0.28 ± 0.01 | 0.06 ± 0.01 | 0.08 ± 0.009 | 0.36 ± 0.09 | 0.02 ± 0.001 | 0.16 ± 0.04 | 0.68 ± 0.06 | 58.7 ± 3.8 | 2.4 ± 0.08 |
|  | STR/Ort | 61.8 ± 8.7 | 25.5 ± 10.4 | 0.30 ± 0.02 | 0.06 ± 0.01 | 0.08 ± 0.01 | 0.44 ± 0.1 | 0.02 ± 0.002 | 0.21 ± 0.06 | 0.61 ± 0.09 | 62.3 ± 3.4 | 2.4 ± 0.05 |
| 6 week | CBA | 50.3 ± 1.4 | 0.13 ± 0.005 | 0.48 ± 0.02 | 37.8 ± 5.0 | 0.13 ± 0.005 | 0.66 ± 0.07 | 0.03 ± 0.003 | 0.26 ± 0.04 | 0.58 ± 0.05 | 47.6 ± 3.6 | 2.3 ± 0.09 |
|  | STR/Ort | 55.5 ± 1.0** | 0.19 ± 0.02* | 0.59 ± 0.03*** | 37.7 ± 2.8 | 0.19 ± 0.02* | 0.79 ± 0.24 | 0.03 ± 0.02 | 0.31 ± 0.11 | 0.59 ± 0.08 | 60.0 ± 3.2* | 2.6 ± 0.07 |
