# Supplementary material for: Endochondral Growth Defect and Deployment of Transient Chondrocyte Behaviors Underlie Osteoarthritis Onset in a Natural Murine Model
Source: Arthritis Rheumatol. 2016 Mar 28;68(4):880–91. doi: 10.1002/art.39508 (PMC4832379; doi:10.1002/art.39508)
Supplement: Supplementary file 5 — Supplementary Information [file ART-68-880-s005.doc]

**Supplementary methods**

*Animals*

Male STR/Ort (bred in-house), and CBA (Charles River, UK) mice were used in all experiments and kept in polypropylene cages, with light/dark 12-h cycles, at 21 ± 2°C, and fed ad libitum with maintenance diet (Special Diet Services, Witham, UK). All procedures complied with Animals (Scientific Procedures) Act 1986 and local ethics committee. STR/Ort mice were maintained by brother/sister pairing.

*Meta-analysis of microarray data*

Microarray data published by us previously (22) was revisited and gene ontology classification was performed using DAVID (Database for Annotation, Visualization, and Integrated Discovery) (<http://david.abcc.ncifcrf.gov/>) (24). Data was originally generated from Affymetrix mouse gene microarray profiling of articular cartilage from affected mice (18–20 week (mature; n=7) and 40–42 (aged; n=14) week old STR/Ort mice and unaffected mice (8–10 (young; n=5), 18–20 (mature; n=5) and 40–42 (aged; n=9) week old CBA mice and 8-10 week old STR/Ort (young; n=6) (22). Analysis was performed on those genes which were previously determined as being significantly up- (n=491) or down-regulated (n=407) (>1.5 fold change) (22).

*RNA extraction*

RNA was extracted from knee joint articular cartilage from mice at ages 8-10, 18-20 and 40+ weeks of age, as described (22). Tibial and femoral surfaces were exposed, articular cartilage from each condyle isolated using a Friedman-Pearson micro-rongeur (extra-fine tip, 0.7-mm cup width; Fine Science Tools) and samples kept in RNAlater (Qiagen, West Sussex, UK). Articular cartilage from STR/Ort and CBA joints (n=3 joints/sample for each strain at each age) was immersed in QIAzol (Qiagen), homogenized and total RNA isolated with a Qiagen Mini Kit according to the manufacturer’s instructions. For each sample, total RNA content was assessed by absorbance at 260 nm and purity by A260/A280 ratios.

*Multiplex RT-qPCR Analysis*

A GeXP multiplex RT-qPCR assay was designed for gene targets; Ank, Dmp1, Enpp1, Mepe, Opn, Phex, and Sost (Suppl. Table 1) (25,26). Target-specific reverse transcription was performed as previously described, in accordance with manufacturer’s instructions (Beckman Coulter, Wycombe, UK) using 50ng of total RNA. Briefly, a reverse transcription master mix was prepared and reactions performed using the programme protocol: 48^o^C, 1min; 42^o^C, 60min and 95^o^C, 5mins. An aliquot from this reaction was mixed with Genomelab kit gene-specific PCR master mix, including Thermo-Start Taq DNA polymerase (Thermoscientific). Following the qPCR step (95^o^C activation step, 10mins, with 35 cycles of 94^o^C, 30secs, 55^o^C, 30secs and 70^o^C, 60secs), products were separated and detected by capillary gel electrophoresis using CEQTM 8000 genetic analysis system. Peaks were matched to the corresponding gene and analysed using GenomeLab fragment analysis software (Beckman Coulter).

*Immunohistochemistry*

Tibiae were dissected, fixed in 70% (v/v) ethanol for 24 hours before being decalcified in 10% EDTA pH 7.4 for approximately 3 weeks at 4^o^C with regular changes. Tissues were dehydrated, embedded in paraffin wax, and coronal sections cut at 6μm. For immunohistochemical analysis, longitudinal sections were dewaxed in xylene and rehydrated. For antigen unmasking, sections were either incubated at 37°C for 30 min in 0.1% trypsin [prior to exposure to anti-sclerostin (R&D systems), anti-MMP13 (Abcam) or anti-Col10a1 (Prof. Boot-Handford, University of Manchester) antibodies] or at 37^o^C for 1 hour in citric acid buffer [before incubation with anti-MEPE antibodies (Prof. Rowe, Kansas University Medical Center)]. Endogenous peroxidases were blocked by treatment with 0.03% H_2_O_2_ in methanol (Sigma). MEPE and MMP13 primary antibodies were used at a dilution of 1/200; sclerostin antibodies at a dilution of 1/100; and Col10a1 antibodies at of 1/500 with appropriate IgG controls used. The Vectastain ABC universal kit (Vector Laboratories, Peterborough) was used according to the manufacturer’s instructions. The sections were dehydrated, counterstained with haematoxylin and mounted in DePeX.

*Articular cartilage and growth plate zone analysis*

Multiple 6µm coronal sections were stained in toluidine blue (Sigma) (0.1% (w/v) in 0.1M solution of acetate buffer, pH 5.6). These stained sections (n>6) from the joints of 4 individual mice (at each age) were used to measure the width of: (i) the uncalcified cartilage, calcified cartilage, and subchondral bone at 10 different points across the joint surface (ii) the proliferating and hypertrophic zones, as well as the total growth plate width, measured at 10 different points along the length of the growth plate.

*Joint imaging by micro-computed tomography (µCT)*

Joints (tibia and femur) were dissected and fixed in neutral buffered formalin (NBF) for 24 hours before being transferred to 70% (v/v) ethanol. The bones were scanned using a laboratory source for 5 micron voxel size and at a synchrotron for 1 micron voxel size.

The laboratory scans were performed with an 1172 x-ray microtomograph (Skyscan, Belgium) to evaluate the cortical and trabecular bone geometry. High-resolution scans with an isotropic voxel size of 5 µm were acquired (55 kV, 0.5 mm aluminium filter, 0.6° rotation angle, 2 frame averaging). The scans were reconstructed using NRecon software (Skyscan, Belgium) and filters were applied to the images prior to reconstruction to remove artifacts, including beam-hardening and ring artifacts. A 1000 µm section of the metaphysis 250 µm off the reference plate was taken for analysis of trabecular bone. The base of the growth plate was used as a standard reference point. A 500 µm metaphysis section of the mid-diaphysis, 3735 µm below the reference plate, was scanned for analysis of cortical structure. Data was analysed with CtAn software (Skyscan). Three-dimensional analyses were performed to determine bone volume/tissue volume, trabecular number, trabecular thickness, and trabecular separation at the distal tibia. Cortical bone was measured at the midshaft region of the bone.

The synchrotron radiation microtomography was performed at Diamond Light Source on the Diamond-Manchester Branchline I13-2 (27) using 19 keV monochromatic x-rays. 1800 projections were collected using a 4x magnification lens by a PCO 4000 CCD imaging camera with 4008 x 2672 pixels, giving an effective pixel size of 1.1 µm. The projections were normalised and reconstructed using a filtered back projection algorithm to produce 3D volumes of the X-ray attenuation (28). A typical 3D reconstruction of the joint and 2D slice through the image volume is shown in Fig. 5A. Each image volume was median filtered to reduce noise and then the tibia was segmented in Avizo (V8.0, VSG, Burlington, USA), using a region-growing algorithm (Fig. 5B). The volume images were aligned along the metaphyseal tibial shaft (defined as the z-axis) and the central point of each individual bony bridge crossing the entire growth plate width was manually identified. Once all the bridges were selected, each was quantified and rendered a separate colour to confirm correct manual identification (Fig. 5C).

To quantify the bone bridge local number density, a method for projecting them onto the joint surface was developed, similar to techniques used for mapping vascular features (29). Each bridge was orthographically projected onto the tibial joint surface using an in-house line intercept method implemented in Matlab (R2012b, Mathworks, USA). The method consists in sending straight lines along the (z) direction passing through the bridges, and by detecting only the lines that intercept the tibial joint surface. After projection, the distribution of the areal density of bridges was calculated and superimposed on the tibial joint surface, allowing location and number to be mapped in a single 3D image (Fig. 5). The areal density, d, is defined as the number of bridges per 256 µm x 256 µm window and the histograms presented here were obtained with a class interval size of ∆d=1.

*Metatarsal organ cultures*

Embryonic metatarsal organ cultures provide a well‐established model of endochondral bone growth (30, 31). Metatarsal bones (E15) were cultured in a humidified atmosphere (37 °C, 5% (v/v) CO2) in 24-well plates for up to 7 days. Each culture well contained 300μl α-minimum essential medium (MEM) supplemented with 0.2% BSA Fraction V; 1 mmol/l β-glycerophosphate (βGP); 0.05mg/ml L-ascorbic acid phosphate; 0.05mg/ml gentamicin and 1.25μg/ml fungizone (Invitrogen, Paisley, UK) as previously described. The total length of the bone through the centre of the mineralising zone was determined using Image J analysis software. The length of the central mineralisation zone was also measured. All results are expressed as a percentage change from harvesting length which was regarded as baseline.

*Sclerostin ELISA*

Serum samples were isolated from CBA and STR/Ort mice at 8-10 weeks, 18-20 weeks, and 40+ weeks following cardiac puncture (n=4 for each strain at each age). Serum sclerostin levels were measured using a mouse/rat sclerostin ELISA kit (R&D systems).
